# Digital Technology Use and Mental Health Consultations: Survey of the Views and Experiences of Clinicians and Young People

**DOI:** 10.2196/44064

**Published:** 2023-04-17

**Authors:** Raphael Rifkin-Zybutz, Nicholas Turner, Jane Derges, Helen Bould, Felicity Sedgewick, Rachael Gooberman-Hill, Myles-Jay Linton, Paul Moran, Lucy Biddle

**Affiliations:** 1 Centre for Academic Mental Health Bristol University Medical School Bristol United Kingdom; 2 Population Health Sciences Bristol University Medical School Bristol United Kingdom; 3 Medical Research Council Integrative Epidemiology Unit University of Bristol Bristol United Kingdom; 4 Gloucestershire Health and Care National Health Service Foundation Trust Gloucester United Kingdom; 5 School of Education University of Bristol Bristol United Kingdom; 6 Bristol University Medical School University of Bristol Bristol United Kingdom; 7 The National Institute for Health Research Applied Research Collaboration West University Hospitals Bristol and Weston National Health Service Foundation Trust Bristol United Kingdom; 8 Biomedical Research Centre University Hospitals Bristol and Weston National Health Service Foundation Trust Bristol United Kingdom

**Keywords:** internet, adolescent, child, mental health, anxiety, patient-physician relationship

## Abstract

**Background:**

Digital technologies play an increasingly important role in the lives of young people and have important effects on their mental health.

**Objective:**

We aimed to explore 3 key areas of the intersection between digital technology and mental health: the views and experiences of young people and clinicians about digital technology and mental health; implementation and barriers to the UK national guidance recommendation—that the discussion of digital technology use should form a core part of mental health assessment; and how digital technology might be used to support existing consultations.

**Methods:**

Two cross-sectional web-based surveys were conducted in 2020 between June and December, with mental health clinicians (n=99) and young people (n=320). Descriptive statistics were used to summarize the proportions. Multilinear regression was used to explore how the answers varied by gender, sexuality, and age. Thematic analysis was used to explore the contents of the extended free-text answers. Anxiety was measured using the Generalized Anxiety Disorder Questionnaire-7 (GAD-7).

**Results:**

Digital technology use was ubiquitous among young people, with positive and negative aspects acknowledged by both clinicians and young people. Negative experiences were common (131/284, 46.1%) and were associated with increased anxiety levels among young people (GAD-7 3.29; 95% CI 1.97-4.61; *P*<.001). Although the discussion of digital technology use was regarded as important by clinicians and acceptable by young people, less than half of clinicians (42/85, 49.4%) routinely asked about the use of digital technology and over a third of young people (48/121, 39.6%) who had received mental health care had never been asked about their digital technology use. The conversations were often experienced as unhelpful. Helpful conversations were characterized by greater depth and exploration of how an individual’s digital technology use related to mental health. Despite most clinicians (59/83, 71.1%) wanting training, very few (21/86, 24.4%) reported receiving training. Clinicians were open to viewing mental health data from apps or social media to help with consultations. Although young people were generally, in theory, comfortable sharing such data with health professionals, when presented with a binary choice, most reported not wanting to share social media (84/117, 71.8%) or app data (67/118, 56.8%) during consultations.

**Conclusions:**

Digital technology use was common, and negative experiences were frequent and associated with anxiety. Over a third of young people were not asked about their digital technology use during mental health consultations, and potentially valuable information about relevant negative experiences on the web was not being captured during consultations. Clinicians would benefit from having access to training to support these discussions with young people. Although young people recognized that app data could be helpful to clinicians, they appeared hesitant to share their own data. This finding suggests that data sharing has barriers that need to be further explored.

## Introduction

### Background

Digital technologies play an increasingly important role in the lives of young people. Survey data from the European Commission show that across Europe, 95% of young people used the internet daily in 2021, compared with 82% in 2012 [1]. Meanwhile, 87% of those aged between 12 and 15 years use social media sites or apps, and over 90% own their own smartphones [2]. Negative experiences on the web have become a common occurrence, with >50% of children reporting having had a negative experience on the web [2]. Therefore, there has been increasing concern about possible negative impacts on mental health, including increased levels of depression and anxiety, loneliness, and social isolation, in addition to exposure to cyberbullying [3-7].

It is also increasingly asserted that asking about digital technology use and its associated risks is an important part of a comprehensive assessment of young people during mental health consultations [8]. Royal College of Psychiatrists (the United Kingdom) guidance, for instance, stipulates that “questions around technology use should become a core part of biopsychosocial assessments and formulations” and highlights the need for research in this area [9]. Currently, little is known about whether this recommendation is being implemented or whether there are barriers preventing such discussions from taking place.

However, there are also positive aspects to the increasing use of digital technology. In principle, digital technology provides resources to support and educate those seeking help [10,11], a function that played a particularly important role during the COVID-19 pandemic [12]. Furthermore, there has been an increasing push to harness digital technology to support mental health, for example, the proliferation of mood-monitoring apps [13]; web-based support services during crises [14]; and novel social media platforms designed to cater for mental health, such as those providing a forum for young people to express their feelings and obtain peer support [15].

Apps provide a digital record of an individual’s health and behavior that, in principle, can be used to help inform routine mental health assessments. Preliminary surveys have shown that among adults in the United States, psychiatric outpatients would generally be willing to share their social media posts with their current therapists [16]. Surveys of clinicians have shown that in practice, they had often viewed patient’s electronic media such as texts and occasionally their social media posts as part of their care and had generally found this helpful [17]. However, none of these studies examined patients aged <18 years.

### Objective

Therefore, we aimed to address these gaps in knowledge by conducting a web-based survey of young people and clinicians. We aimed to investigate the following three main areas of interest: (1) clinicians’ and young people’s attitudes toward and experiences of the digital world in relation to mental health, (2) the discussion of digital technology use by young people and clinicians during mental health consultations, and (3) the use of digital technology to aid consultations (including the potential to use novel data streams to inform the consultation).

## Methods

We conducted 2 cross-sectional web-based surveys: 1 with mental health clinicians and 1 with young people.

### Ethics Approval

Research ethics approval was provided by the Health Sciences Research Ethics Committee at the University of Bristol (references: 103102; 104403).

### Recruitment

We recruited young people aged between 14 and 24 years from the general population. Any young person was eligible to take part, irrespective of their mental health status or experience of service use. Upon clicking the survey link, potential participants were taken to a study information sheet and were required to provide e-consent before starting to complete the survey. Parental assent was obtained for participants aged <16 years.

Eligible clinicians were health or social care workers working in the statutory or nonstatutory sector, who spent at least a proportion of their working time supporting the mental health needs of young people aged ≤24 years. Both surveys were conducted in the United Kingdom, but international participants were eligible to participate.

Convenience sampling was used, in which participants were recruited through public advertisements circulated via professional social media accounts, tagging relevant organizations such as mental health charities, youth groups, and professional bodies. The advertisements were “re-tweeted,” creating a web-based snowballing. We also approached local schools, clinical networks, university services, and national charities directly through email. Furthermore, an advertisement was placed on the UK-based young person’s mental health app (“Tellmi”).

### Data Collection

Each survey consisted of 1 questionnaire, with complementary versions for clinicians and young people. These were hosted on SurveyMonkey for 4 months in 2020 (clinician survey: June to September; young person survey: August to December).

The clinician and young people questionnaires followed the same format and covered identical topic areas. The majority of questions were fixed choice or Likert-scale questions were used, with a smaller number of open-ended questions to provide additional qualitative insights. The full versions of both questionnaires can be found in Multimedia Appendices 1 and 2. The main topic areas included views on the effect of digital technology use on mental health, experiences of discussing digital technology use during mental health consultations, and experiences of using digital technology to help facilitate mental health consultations. In addition, young people were asked to complete the Generalized Anxiety Disorder Questionnaire 7 (GAD-7) to assess current anxiety symptoms. The GAD-7 has been validated for use in this age range [18]. Clinicians were asked about their job roles, experiences, age of young people they supported, country of practice, and length of practice.

A draft copy of the young people questionnaire was shared with a young person’s advisory group panel and a further group of neurodiverse young people who offered advice on acceptability, clarity, and ease of completion. Final amendments were made to address feedback before the finalized survey was administered. The clinician survey questionnaire was piloted with a small number of clinical colleagues to refine the final draft.

Upon completion, participants of both surveys could opt to enter into a prize draw. Signposting information for mental health services was also provided at the end of the young people questionnaire for participants who wanted further support.

### Analysis

#### Quantitative Data

Quantitative data were analyzed using Stata (version 17; StataCorp) [19]. The clinician and young person surveys were analyzed separately. Survey questions varied in design, although most were statements that were linked to the choice of the following 6 options: “Strongly Disagree,” “Disagree,” “Neutral,” “Agree,” “Strongly Agree,” and “Unsure.” For the purposes of analysis, these were combined into positive (agree and strongly agree), neutral (neutral and unsure), or negative (disagree and strongly disagree) categories. All other questions were statements that offered categorical responses (such as “This has Happened,” “I would like this to happen,” and “I would not like this to happen”), which were analyzed as categorical variables. Descriptive statistics were used to summarize the proportions.

Some questions were restricted to subgroups of the survey; for example, the questions asking young people about their experiences when receiving help with their mental health were only asked to those who reported receiving such help. Fisher exact test was used to assess differential completion rates across gender and sexuality, and logistic regression was used to assess differential completion rates across age groups.

For the young people questionnaire, exploratory analyses were performed to determine whether answers to key questions varied by demographic background. Ordinal logistic regression was used to examine whether responses varied by age, gender, and reported sexuality. The proportional odds assumption was assessed using the Brant test. Because of the low number of individuals reporting gender other than male or female, analyses checking whether responses varied across gender examined only the differences between those reporting male or female. Age was recoded into groups of people aged <16, 16 to 18, and ≥18 years. Our reference category was the most common response in each group, which was female, age <16 years, and heterosexual. Given the low numbers for most ethnic minority groups, we could not meaningfully examine how experiences or views differed across ethnicities. Demographic data were not collected in the clinician questionnaires.

Linear regression was used to assess the association between total GAD-7 scores and previous negative web-based experiences. For the purposes of this analysis, we recoded answers to the question, “I have had bad experiences online that have affected my mental health” into a binary variable of yes (“strongly agree” or “agree”) or no or unsure (“Neutral,” “Disagree,” “Strongly Disagree,” or “Unsure”). A simple unadjusted regression was used followed by a multiple linear regression, which was adjusted for possible demographic confounders including age, gender, sexuality, ethnicity, and a history of being treated for a mental health condition.

#### Qualitative Data

Thematic analysis was used to explore the contents of the extended free-text answers. An open inductive approach was used in which codes were derived from the data and were used to label the text according to the meaning expressed. Coding was undertaken collaboratively by LB and JD. All individual codes were displayed on a code list and then organized into thematic categories by LB. The content of each theme was then examined by retrieving all relevant labeled data.

## Results

### Participant Demographics

A total of 320 young people completed the survey. The sample consisted of participants who are mainly White British (271/320, 84.7%), of female sex (196/320, 61.3%), and heterosexual (214/320, 67.1%). More than one-third of the participants (121/320, 437.8%) had received help for mental health difficulties. Smartphone ownership (295/320, 92.2%) and the use of social media (289/320, 90.3%) were almost ubiquitous.

Furthermore, 99 clinicians completed the survey. Most of them had lengthy clinical experience (median of 12 years), were based in the United Kingdom (88/99, 88.9%), and worked in the statutory sector (64/99, 64.7%). They were recruited from a wide variety of roles, of which psychologists (28/99, 28.3%), psychiatrists (23/99, 23.2%), and other (11/99, 11.1%) were the most common. The “other” category was composed of a diverse range of associated professionals including nursing assistants, well-being workers, advisers, and counselors.

Full demographic information for both surveys can be found in Tables 1 and 2.

**Table 1:** Young people questionnaire demographics (n=320).

Characteristics			Participants, n (%)
**Age (years)^a^**			
	14-15	132 (41.3)	
	16-17	82 (25.6)	
	18-25	106 (33.1)	
	Missing	0 (0)	
**Ethnicity**			
	White	271 (84.7)	
	Black, African, Caribbean, or Black British	5 (1.6)	
	Asian or Asian British	16 (5)	
	Mixed or Multiple ethnic groups	21 (6.6)	
	Other ethnic group	7 (2.2)	
	Missing	0 (0)	
**Sexuality**			
	Heterosexual	214 (67.1)	
	LGB+^b^	97 (30.4)	
	Prefer not to say	8 (2.5)	
	Missing	1 (0.3)	
**Gender**			
	Female	196 (61.3)	
	Male	110 (34.4)	
	Any other response (including nonbinary)	12 (3.8)	
	Prefer not to say	2 (0.6)	
	Missing	0 (0)	
**Current occupation**			
	Any education	264 (82.5)	
	Any employment	121 (37.8)	
	NEET^c^	19 (6)	
	Missing	0 (0)	
**Ever received help for a mental health problem**			
	Yes	121 (37.8)	
	No	148 (46.4)	
	Missing	51 (16.6)	
**Device ownership**			
	Smartphone	295 (92.2)	
	Laptop or desktop	275 (85.9)	
	Tablet	112 (35)	
	Missing	20 (6.2)	
**Use of social media**			
	Yes	289 (90.3)	
	No	10 (3.1)	
	Missing	21 (6.6)	

^a^Median (IQR): 16.9 (15.1-19.8) years.

^b^LGB+: lesbian, gay, and bisexual.

^c^NEET: Not In education, employment, or training.

**Table 2:** Clinician demographics (n=99).

Characteristics		Value
**Sector, n (%)**		
	Statutory	64 (64.7)
	Multiple	4 (4)
	Charitable	15 (15.2)
	Private	10 (10.1)
	Education	5 (5.1)
	Other	1 (1)
**Job role, n (%)**		
	Psychiatrist	23 (23.2)
	Nurse	8 (8.1)
	Psychologist	28 (28.3)
	Social worker	3 (3)
	GP^a^	3 (3)
	Student well-being support	10 (10.1)
	Manager	4 (4)
	Practitioner	9 (9.1)
	Other	11 (11.1)
**Age range of young people supported (years), n (%)**		
	<12	54 (54.5)
	12-15	61 (61.6)
	16-17	72 (72.7)
	18-24	46 (46)
**Country of practice, n (%)**		
	The United Kingdom	88 (88.9)
	Europe (excluding the United Kingdom)	5 (5.1)
	Rest of world	5 (5.1)
	Missing	1 (1)
Length of practice (years), median (IQR)		12 (5.9-20)

^a^GP: general practitioner.

### Views and Experiences of Digital Technology Use in Relation to Mental Well-Being

We explored young people’s use of digital technology in relation to mental health and young people’s and practitioners’ views about the impact of digital technology on mental health. Table 3 presents a summary of the most important data quoted in this section. The full data for all the questions in this section are displayed in Table S1 in Multimedia Appendix 3.

Just over half of the young person sample (157/297, 52.9%) reported having used digital technology or web-based resources to support their mental well-being. Of the 157 young people, 128 (81.5%) answered an open-ended question regarding the resources they had used to do this. These responses were coded into the following 5 categories: formal treatment (17/128, 13.3%), talking to others (32/128, 25%), apps (34/128, 26.6%), psychoeducation (40/128, 31.2%), and distraction (50/128, 39.1%).

Most clinicians agreed that digital technology use could have harmful (91/95, 96%) and helpful (78/95, 82%) effects on young people’s mental health. However, although many young people agreed that social media could be harmful (122/284, 43%) and helpful (102/284, 36%) to mental health, similar proportions remained neutral or unsure about whether social media was harmful (101/284, 36%) or helpful (105/284, 37%).

Exploratory analyses of demographics suggested that compared with female young people, male young people were less likely to agree that social media was harmful to their mental health (odds ratio [OR] 0.31, 95% CI 0.19-0.52; *P*<.001) and more likely to agree that social media had been helpful for their mental health (OR 2.19, 95% CI 1.34-3.58; *P*=.001). Age was associated with views about the harms of social media (*χ*^2^_2_=16.7, *P*<.001) and whether it could be helpful (*χ*^2^_2_=9.5, *P*=.009), with older individuals having more skeptical views of social media. Compared with young people aged <16 years, both those aged 16 to 18 years (OR 2.36, 95% CI 1.32-4.25; *P*=.004) and >18 years (OR 3.67, 95% CI 1.31-4.25; *P*<.001) were more likely to agree that social media was harmful to their mental health. Compared with young people aged <16 years, those aged >18 years were less likely to agree that social media had been helpful for their mental health (OR 0.49, 95% CI 0.29-0.84; *P*=.009). There was no evidence that young people who identified as lesbian, gay, and bisexual (LGB+) had different views on the harms of social media (*P*=.31; Table S2 in Multimedia Appendix 3), but they were more likely to agree that social media had been beneficial for their mental health (OR 2.24, 95% CI 1.33-3.75; *P*=.002).

A large proportion of young people reported having bad web-based experiences, which affected their mental health (131/283, 46.3%). Male young people were less likely to report having these experiences than female young people (OR 0.56, 95% CI 0.34-0.91; *P*=.02). There was no evidence that the likelihood of reporting these experiences varied by age (*P*=.17) or sexuality (*P*=.12; Table S2 in Multimedia Appendix 3).

Within the linear regression analysis, reporting of bad experiences on the web was significantly associated with higher scores on the GAD-7 at the time of the survey (3.26, 95% CI 1.93-4.58; *P*<.001). This association attenuated but remained after adjusting for age, gender, sexuality, and a history of being treated for a mental health condition (1.93, 95% CI 0.72-3.14; *P*=.002).

**Table 3:** General attitudes and experiences of digital technology.

Questions		Participant sample	Participants, n/N (%)				Missing participants, n/N (%)
			Agree	Unsure or neutral	Disagree		
**Social media helpful or harmful?**							
	Social media has been helpful to my mental health	YP^a^	102/284 (35.9)	105/284 (37)	77/284 (27.1)	36/320 (11.3)	
	Young people’s digital technology use can be helpful to their mental health	C^b^	78/95 (82.1)	15/95 (15.8)	2/95 (2.1)	4/99 (4)	
	Social media has been harmful to my mental health	YP	122/284 (43)	101/284 (35.6)	61/284 (21.5)	36/320 (11.3)	
	Young people’s digital technology use can be harmful to their mental health	C	91/95 (95.8)	4/95 (4.2)	0/95 (0)	4/99 (4)	
**Bad experiences on the web**							
	I have had bad experiences online that have affected my mental health	YP	131/283 (46.3)	59/283 (20.9)	93/283 (32.9)	37/320 (11.6)	

^a^YP: young person (n=320).

^b^C: clinician (n=99).

### Discussing Digital Technology Use During Mental Health Consultations

We explored whether practitioners ask young people about their digital technology use and its possible impacts on their well-being when an individual presents with mental health difficulties. A summary of the most important data quoted in this section can be found in Tables 4 and 5, and the full data for all the questions in this section are displayed in Table S3 in Multimedia Appendix 3.

Most clinicians (61/83, 74%) agreed that exploring digital technology use should form an essential part of mental health risk assessment. This was also generally acceptable to young people in the survey, most of whom (177/265, 66.8%) agreed, “I would find it okay to discuss my digital technology use with a health professional.” There was no evidence of differences in views between genders (*P*=.70) or across different sexualities (*P*>.27; Table S2 in Multimedia Appendix 3). There was a trend toward views differing by age (*χ*^2^_2_=5.3, *P*=.07), but these results were not statistically significant. Compared with young people aged <16 years, those aged >18 years (OR 2.05, 95% CI 1.09-3.88) were more likely to find it acceptable to discuss their digital technology use.

Although most clinicians (63/84, 75%) reported feeling confident talking to young people about their digital technology use, a large proportion of young people (111/264, 42.1%) felt that health professionals did not understand the nature of their behavior on the web. Despite a high level of confidence, most clinicians (59/83, 71%) also reported wanting training on how to discuss digital technology use in a health care setting. Notably, very few health care professionals reported having received any such training or guidance (21/86, 24%) or access to a protocol (6/86, 7%) to help guide discussions in this area.

Despite recognizing the importance of the discussion, only about half of the clinicians (48/86, 56%) reported that asking about digital technology use was integrated into their assessments, and just under half (42/85, 49%) reported asking about digital technology use routinely. Most others (34/85, 40%) only occasionally discussed the topic. This did not appear to differ across job roles (Fisher exact test, *P*=.70). There was concordance with this finding in our young person survey, in which 48 (39.6%) of the 121 young people who reported that they had received mental health help had never been asked about their digital technology use by any professional. Although gender was not associated with the likelihood of being asked about digital technology use (*P*=.23; Table S2 in Multimedia Appendix 3), age was associated (*χ*^2^_2_=8.4, *P*=.02), with those aged >18 years being much less likely to have been asked compared with those aged <16 years (OR 0.22, 95% CI 0.07-0.67; *P*=.008). There was also some evidence that young people who identified as LGB+ were more likely to be asked (OR 2.32, 95% CI 1.00-5.39; *P*=.05).

**Table 4:** Discussing digital technology use during mental health consultations: role and knowledge of clinicians.

Questions			Participant sample		Participants, n/N (%)						Missing participants, n/N (%)
					Agree		Unsure or neutral	Disagree			
**The role of health professionals**											
	I would find it okay to discuss digital tech with health professionals	YP^a^		177/265 (66.8)		59/265 (22.3)		29/265 (10.9)	55/320 (17.2)		
	Exploring Digital Technology should form an essential part of mental health consultation	C^b^		61/83 (73.5)		18/83 (21.7)		4/83 (4.8)	16/99 (16.2)		
**Health professionals:** **understanding the world on the web**											
	Most health professionals do not understand the way young people use the online world	YP		111/264 (42.1)		115/264 (43.6)		38/264 (14.4)	56/320 (17.5)		
	Overall, I have a good understanding of how young people use digital technology	C		73/95 (76.8)		15/95 (15.8)		7/95 (7.4)	4/99 (4)		
	I would like training in how to talk to young people about their digital technology use	C		59/83 (71.1)		21/83 (25.3)		3/83 (3.6)	16/99 (16.2)		

^a^YP: young person.

^b^C: clinician.

**Table 5:** Discussing digital technology use during mental health consultations: implementation.

Questions				Participants, n/N (%)
**Clinician**				
	**How often do you discuss digital technology use with young people**			
		Routinely		42/85 (49.4)
		Occasionally		34/85 (40)
		Rarely		2/85 (2.4)
		Never		7/85 (8.2)
		Missing		14/99 (14.1)
	**Have you received any training or guidance for talking to young people about their digital technology use?**			
		Yes	21/86 (24.4)	
		No	65/86 (75.6)	
		Missing	13/99 (13.1)	
**Young person**				
	**Have you ever been asked about your digital technology use?**			
		Yes		73/121 (60.3)
		No		48/121 (39.7)
		Missing		51/172 (29.7)

Of the 121 individuals who reported receiving help with their mental health, 67 (55.4%) reported having a bad experience on the web that affected their mental health. Of them, over a third (25/67, 37%) had never been asked about their digital technology use and so had not had their relevant negative experiences on the web explored during their mental health contacts.

When asked about potential barriers to discussing digital technology use, clinicians were most likely to identify none (35/84, 42%), lack of time (29/84, 35%), and lack of knowledge (24/84, 29%). It is also notable that around a fifth (18/84, 21%) reported worrying that young people would be unwilling to have a conversation about their digital behavior.

Survey respondents were asked to describe the topics covered during conversations on the use of digital technology. The 3 most reported topics among young people were the use of social media (52/73, 71%), available mental health apps (49/73, 67%), and website signposts to help resources (43/73, 59%). Clinicians reported a very similar result, with the most discussed topics being social media (71/77, 92%), mental health apps (66/77, 86%), website signposting (60/77, 78%), and discussions about negative experiences on the web (60/77, 78%). Overall, clinicians reported asking more often about topics than young people recalled being asked.

### Discussing Digital Technology Use During Mental Health Consultations—Qualitative Findings

Young people in the survey who had been asked by their clinician about their digital technology use in the context of a mental health consultation were asked whether this conversation had been helpful and to explain their answers in free texts. Of the 58 young people who provided a response, 21 (36%) reported the conversation as helpful, 34 (58%) said it was not helpful, and 3 (5%) were unsure.

Conversations about digital technology use were considered helpful when they allowed young people to talk through a negative experience on the web or to gain insights into how web-based content or behaviors could have a negative effect on their mental health. Some participants indicated that such conversations sometimes meant that they learned how to behave more safely on the web, and 1 stated that they had never had the opportunity to discuss their experiences on the web elsewhere:

It was helpful to break down exactly what I look at on social media and how it impacts my day-to-day life as well as my outlook on society

It was helpful because I’d rather talk to someone about it if I’m struggling with stigma or bullying on social media than attempting to harm myself/end my life

Another participant thought that the conversation about their digital behavior had facilitated greater insights and shared an understanding about their difficulties:

It helped me identify what was harmful to me at the time…I’m glad that I did talk about it as the professionals working within my care were good at responding to that and understood a lot more about me and why I felt the way I did, partly because of the content I was viewing at the time.

However, simply receiving signposts to mental health apps or help sites was generally not considered a useful outcome and could be perceived as dismissive:

[Clinician] did not personally help me and immediately told me to use an app or visit websites to look for help by myself

It was not particularly helpful as my phone does not have space to download mental health apps. We also did not talk enough about the impact of social media and negative experiences online on mental health.

The most commonly reported reason for finding conversations unhelpful was that the clinician conveyed a “negative view” of young people’s digital technology use or appeared not to understand it, which could result in the young person feeling judged or blamed:

It was not helpful because I felt like they were judging me for my use of social media and [saw] social media as something that is always bad.

Digital technology seemed frowned upon:

‘Oh, this is what’s causing your anxiety. You’re too glued to your computer…’ It wasn’t helpful because usage seems frowned upon and is treated insensitively

Two participants found conversation about their use of technology to be intrusive, whereas 4 considered such discussions irrelevant to them (and therefore unhelpful) because they regarded their digital technology use as unproblematic.

### Can Digital Technology Be Used to Assist the Mental Health Consultation?

Our third research area explored whether digital technology could be used as a tool to enhance mental health consultations with young people. Full results of this section are presented in Table S4 in Multimedia Appendix 3.

Most of the clinicians (83/91, 91%) surveyed had used digital technology to support patients. When integrating digital technology into mental health care, clinicians in this survey mostly reported “Prescribing a self-care or mood-monitoring app” (52/87, 60%), meeting with young people via web before face-to-face consultations (31/86, 36%), and offering therapy to be completely on the web (26/87, 30%). Interestingly, although most young people had either experienced or would like to experience being recommended a web-based resource (105/119, 88.2%) or an app (104/118, 88.1%), most did not want web-based appointments either before the first face-to-face meeting (66/119, 55.5%) or generally (71/118, 60.2%).

The survey also explored how novel data streams might be used to help inform mental health consultations, for example, by inviting young people to share mental health apps or social media information with their clinicians. Most clinicians (80/87, 92%) had not asked young people to share their app data with them, but most (63/87, 72%) would consider doing so. This was true for history taking, triaging, risk assessment, and monitoring. Most clinicians (54/83, 65%) thought that having access to app data would be useful to them, with the rest (25/83, 30%) either neutral or unsure.

Most young people (170/264, 64.4%) reported that they would feel comfortable about sharing their mental health app data with a health professional. Young people who had received mental health care had generally not been asked to share data either from social media (107/117, 91.4%) or a mental health app such as mood-monitoring app (106/118, 89.8%). Despite reporting feeling comfortable with sharing data in principle, most young people did not want this to form a part of the mental health consultation, with most of young people (84/117, 71.8%) going on to state that they would not like to share data posted on social media, and a smaller majority (67/118, 56.8%) not wanting to share data from a mental health–specific app. Compared with young people aged <16 years, those aged >18 years were much more likely to want to be asked to share data from mental health apps (OR 10.51, 95% CI 2.48-44.48; *P*=.001), and there was some evidence that they were more likely to want to share posts on social media (OR 3.28, 95% CI 0.82-13.09; *P*=.09). Male participants were more likely to want to be asked to share posts made on social media (OR 3.53, 95% CI 1.06-11.80; *P*=.04), but there was no evidence of a change in attitude toward sharing app data (*P*=.49; Table S2 in Multimedia Appendix 3). There was no evidence of differing views by sexuality (*P*=.78; Table S2 in Multimedia Appendix 3).

## Discussion

### Principal Findings

Our study encompassed views and experiences about digital technology use and its relevance to mental health consultations among both young people and clinicians involved in delivering mental health services to children, adolescents, and young people. To our knowledge, this is the first study to explore the frequency of discussions about digital technology during mental health consultations from the perspectives of both professionals and young people.

### Views and Experiences of Digital Technology Use in Relation to Mental Well-being

Our results align with prior research demonstrating that most young people now own devices that give them instant access to the web [2]. In concordance with previous research, the participants in our study often reported that their experiences on the web affected their mental health, and there was evidence that female participants were more likely to report having had such experiences [20,21]. Therefore, it follows that female young people were more likely to view social media as harmful and less likely to appraise it as helpful. This aligns with previous work that has also suggested that women might be more susceptible to the negative effects of social media [22]. The mixed impacts of digital technology use were also recognized by clinicians.

LGB+ youth were more likely to feel that social media could be helpful and less likely to think of social media as harmful. This could be explained by previous findings that web space is especially important for LGB+ individuals to find like-minded individuals and seek support from friends on the web [23]. However, the same research also identified that LGB+ individuals are more frequently abused on the web. Our sample size may have been too small to demonstrate the same effect [23].

Furthermore, we showed that even after adjusting for gender, sexuality, ethnicity, and mental health history, the reporting of these experiences was associated with higher levels of anxiety, suggesting possible important and enduring effects. This aligns with prior work showing that negative experiences on the web such as cyberbullying are linked to poor mental health outcomes, although this may be true for a wider range of experiences [24]. However, it is important to note that the cross-sectional nature of the analysis means that we are not able to establish the direction of causation, and these findings could also indicate that people with higher anxiety are more likely to have negative experiences on the web or to recall and report negative experiences on the web. Longitudinal studies are needed to further investigate this.

### Discussing Digital Technology Use During Mental Health Consultations

Despite clinicians generally agreeing that enquiry about a young person’s use of digital technology should form an essential part of routine mental health consultations [9], we also found that clinicians asked about digital technology use in an ad hoc manner rather than routinely. This leads to a considerable proportion of young people not having this area explored in mental health contexts. Indeed, around 40% of those who have had adverse experiences on the web that they recognized as affecting their mental health did not have these explored during mental health consultations, suggesting that this is an important area of deficit. The main barriers to discussion appeared to be time, knowledge, and lack of expertise, with most clinicians lacking specific training or protocols to help guide them.

Although our qualitative data indicate good potential for conversations to be beneficial, this lack of guidance may explain why over half of the young people in our survey who had been asked about digital technology use by their clinicians described the conversation as unhelpful. Some young people have also reported experiencing negative attitudes from practitioners regarding social media. Such experiences may underlie the belief expressed by >40% of young people that clinicians do not understand how young people engage with the web, despite most clinicians believing otherwise. This schism of opinion points to a gap in understanding, which we explored in greater depth in subsequent qualitative research and described elsewhere [25]. Similarly, the belief that young people would be unwilling to discuss their behavior on the web was also cited by some clinicians as a barrier to asking; nevertheless, two-thirds of the young people in our survey agreed that they would find it acceptable for a clinician to raise this topic, and indeed those who had been asked wished to explore this in some depth.

To address the need for guidance, a recent study by our group [26] used the Delphi format to investigate good practice indicators for discussions about digital technology use between young people and clinical professionals. This study highlighted the need for discussions to move beyond information gathering to take the form of a deeper conversation with a focus on encouraging individuals to reflect on the meanings and impact of their behavior on the web. This builds on the findings reported here, in which young people clearly indicated that discussion is unhelpful if it is merely focused on signposting to help resources. Given the expressed desire for training and guidance and that time is identified as a barrier to having these discussions, research such as this that seeks to guide and suggest areas of focus may be of help to professionals working with young people.

### Can Digital Technology Be Used to Assist the Mental Health Consultation?

In terms of using digital technology to augment mental health conversations, most clinicians reported that digital technology has become routinely involved in the delivery of services, including meeting young people on the web as well as recommending digital interventions such as apps or helpful websites. This may be partially because of changing practice since the COVID-19 pandemic, which caused a pivot to increased use of digital technology to deliver remote therapy [27]. However, although previous studies have shown a general acceptability of web-based meetings [28], our results suggest that most young people would not want web-based meetings to replace face-to-face consultations. This agrees with previous research suggesting that mental health professionals see technology as primarily a preventative or psychoeducational tool rather than a replacement for face-to-face therapy [29]. However, other studies have suggested that there is a minority of individuals who would prefer web-based to face-to-face therapy [28], which aligns with our findings that around a fifth of young people would prefer to meet on the web rather than face to face, and around a third favor meeting on the web first before meeting in person. Research capturing young people’s experiences of remote provision during COVID-19 is limited to date but may shed further light going forward [30]. It seems likely that there is a mix of needs within the population that may require flexible service provision.

Clinicians reported enthusiasm for the prospect of potentially integrating data from young people’s mood-monitoring apps or social media to enable better assessments, and young people overall felt comfortable sharing data with clinicians. This fits with other preliminary research in this area [31]. However, young people remained skeptical of the idea of this happening during mental health consultations, with the majority reporting that they would not want this when presented with a binary choice. In addition, there was much greater reluctance to share social media data than mental health app data. This possibly represents the fact that social media information is less curated and viewed as a personal sphere separate from the mental health consultation. In addition, the aforementioned concern expressed by many young people that clinicians do not understand how young people use the web is likely to be of considerable relevance here. Sharing personal data would demand a strong sense of trust, but low confidence in clinician understanding is likely to erode this while also heightening the fear of judgment. Methods to facilitate data sharing, including strategies to remove such barriers, warrant further investigation.

### Strengths and Limitations

This research constituted an in-depth survey of a group of both young people and clinicians. This enabled a comparison between the 2 perspectives. Furthermore, the use of both quantitative and qualitative approaches enabled a more detailed contextualization of some of the quantitative findings. The surveys addressed a recommendation specified by the Royal College of Psychiatrists (the United Kingdom) and is to our knowledge the first piece of research to investigate the uptake of this advice. In addition, the collection of both gender and sexuality information for the young people enabled us to explore how views differed across these demographics**.**

Some limitations of this study are that the sample was self-selected and is likely to be biased toward practitioners and young people with a particular interest in digital technology and the web. On this basis, it is possible that the practitioners included in the study were those most likely to ask young people about their digital technology use and were most willing to consider integrating digital technologies in the consultations. The actual prevalence may in fact be lower. It is also possible that the discussion of digital technology has increased since our data were collected, although evidence from our ongoing work does not support this [26]. Further limitations are the lack of ethnic diversity in the sample of young people; most individuals responding to the survey were White British, although the study did include a large number of individuals with a high proportion of those who identified as LGB+ and with prior experience of mental health issues. The low diversity meant that we were unable to compare the results across different ethnicities. As with all cross-sectional studies, we cannot draw any conclusions on causation for links found, for example, between negative experiences on the web and anxiety.

It is also important to note that the survey was conducted in 2020 in the context of the COVID-19 pandemic, during which professionals’ attitudes and approaches to digital technology had to shift rapidly to accommodate services as face-to-face provision was not possible. Although we asked individuals to report their prepandemic practice, it is likely that some of the attitudes reflected changes in practice that had occurred because of the pandemic. Most practitioners were required to use digital technology to assist consultations during this time, which could have inflated the proportion of reporting meeting young people through the web before face-to-face consultation and providing web-based therapy. Concerns about overuse of or reliance on social media among young people may also have been heightened [32,33]. The pandemic is likely to have changed the way that services operate and accelerated the digitization of some mental health services, which may only be partially captured by these data.

### Conclusions

This is the first study to have simultaneously gathered information from clinicians and young people about digital technology use and how it is explored during mental health consultations. Over a third of young people were not asked about their digital technology use during mental health consultations, and therefore the clinicians missed valuable information including relevant negative experiences on the web impacting mental well-being.

Clinicians are currently operating in this space with little specific training, guidance, or protocols. Almost a third cited a lack of knowledge as a barrier to discussion, and 71% (59/83) reported that they would like training in how to talk to young people about their digital technology use. Therefore, important next steps include supporting clinicians by developing and disseminating effective and efficient methods for managing these important conversations with young people. Particularly notable is that despite the demand for training, over three-quarters of the clinicians also thought they had “a good understanding” of how young people use digital technology. This confidence may be misplaced because it was not shared by young people. This highlights a potential communication and understanding gap and, in turn, a need for knowledge exchange covering fundamental issues regarding the meaning of the web within young people’s lives and in relation to their mental health. Successfully addressing this gap is essential to the viability of open communication and the potential for clinicians to capitalize on digital technology use as a means for enhancing mental health care.

Currently, when discussions about digital technology use happen, young people often regard them as unhelpful because they can feel dismissed, judged, or are merely signposted to web-based help sites or apps. Conversations were helpful when the young person had the opportunity to explore the impact of their digital technology use and when such use was used as a lens to better understand mental health problems more generally. Finding spaces where clinicians can have more time to discuss digital technology in greater depth is likely important given that these conversations are likely to take longer, but lack of time is identified as a barrier to these discussions by clinicians.

## Appendix

Multimedia Appendix 1 Full survey for young people.

Multimedia Appendix 2 Full survey for clinicians.

Multimedia Appendix 3 Full details of results and regression analyses.

## Abbreviations

GAD-7: Generalized Anxiety Disorder Questionnaire-7
LGB+: lesbian, gay, and bisexual
OR: odds ratio
