# Supplementary material for: Digital Technology Use and Mental Health Consultations: Survey of the Views and Experiences of Clinicians and Young People
Source: JMIR Ment Health. 2023 Apr 17;10:e44064. doi: 10.2196/44064 (PMC10152330; doi:10.2196/44064)
Supplement: Multimedia Appendix 1 [file mental_v10i1e44064_app1.pdf]

Adolescence, Digital Technology and Mental Health: young people survey

Participant Information and Consent Statement

What is the purpose of the study?

We know digital technology, like social media and apps can be good and bad for mental health. While some young people may experience problems such as cyberbullying or find unhelpful content online, others like to talk to peers or use apps to help them cope. It has also been suggested that doctors, counsellors and other professionals who support young people with their mental health might be able to look after them better if they talked to the young person about their digital technology use. In this survey, we would like to find out the different ways that young people use digital technology to support their mental health, and their opinions about discussing online experiences and sharing app data with health professionals (e.g. doctors).

Before completing this survey, please read the participant information sheet which can be found here

* 1. Before beginning, please tick to confirm the following:

- [ ] I am aged between 14 and 24 years old
- [ ] I have read the survey information sheet
- [ ] I agree to take part in this survey and for my anonymised data to be processed for research purposes by the University of Bristol

2. For participants aged 14-17 only

- [ ] I am under 18 and I have shown my parent/ carer the information sheet
- [ ] I am the parent/ carer of this child who is under 16 and I consent to them taking part

If you have any questions, please contact Dr Lucy Biddle: lucy.biddle@bristol.ac.uk, or Dr Jane Derges: jane.derges@bristol.ac.uk

Adolescence, Digital Technology and Mental Health: young people survey

About You

3. What is your age?

Years __________

Months __________

4. How would you identify your gender?

- Male
- Female
- Transgender
- Gender Fluid
- Prefer to Self-describe

5. Who do you live with?

- Parent or carer/other family member
- Partner/Spouse
- Friend(s)
- In student accommodation (eg hall of residence)
- Alone

Other (please specify)

6. What is your ethnic group? Choose one option that best describes your ethnic group or background.

- ◯ **White** - English/Welsh/Scottish/Northern Irish/British
- ◯ **White** - Irish
- ◯ **White** - Gypsy or Irish Traveller
- ◯ **White** - Any other White background, please describe below
- ◯ **Mixed/Multiple ethnic groups** - White and Black Caribbean
- ◯ **Mixed/Multiple ethnic groups** - White and Black African
- ◯ **Mixed/Multiple ethnic groups** - White and Asian
- ◯ **Mixed/Multiple ethnic groups** - Any other Mixed/Multiple ethnic background, please describe below
- ◯ **Asian/Asian British** - Indian
- ◯ **Asian/Asian British** - Pakistani
- ◯ **Asian/Asian British** - Bangladeshi
- ◯ **Asian/Asian British** - Chinese
- ◯ **Asian/Asian British** - Any other Asian background, please describe below
- ◯ **Black/ African/Caribbean/Black British** - African
- ◯ **Black/ African/Caribbean/Black British** - Caribbean
- ◯ **Black/ African/Caribbean/Black British** - Any other Black/African/Caribbean background, please describe below
- ◯ **Other ethnic group** - Arab
- ◯ **Other ethnic group** - Any other ethnic group, please describe below

Other - describe here

____

7. How would you identify your sexuality?

- ◯ Bisexual
- ◯ Gay man/woman
- ◯ Heterosexual/straight
- ◯ Not sure
- ◯ Prefer not to say
- ◯ Prefer to Self-describe

____

8. Are you currently (at school or 6th form college or university) enrolled on any full-time or part-time education course?

- ◯ Yes
- ◯ No

9. Do you do any paid work (either as an employee or casual part-time work)?

- Yes
- No

10. Which country are you located in?

- England
- Wales
- Scotland
- Northern Ireland
- Other (please specify)

Adolescence, Digital Technology and Mental Health: young people survey

About your health

11. In general, how would you rate your mental health?

- Very poor
- Poor
- Okay
- Mostly good
- Excellent

12. Over the last 2 weeks how often have you been bothered by the following problems?

	Not at all	Several days	More than half the days	Nearly every day
Feeling nervous, anxious, on edge	◯	◯	◯	◯
Not being able to stop or control worrying	◯	◯	◯	◯
Worrying too much about different things	◯	◯	◯	◯
Having trouble relaxing	◯	◯	◯	◯
Being so restless that it is hard to sit still	◯	◯	◯	◯
Becoming increasingly annoyed or irritable	◯	◯	◯	◯
Feeling afraid, as if something awful might happen	◯	◯	◯	◯

13. Have you ever been treated for a mental health problem (eg. depression, anxiety, eating disorder)?

◯ Yes

◯ No

If no, please go to question 16

14. If yes, can you tell us what this was?

[ ]

15. Have you been diagnosed with any of the following? (Tick all that apply)

☐ ADHD

☐ Autism spectrum disorder

☐ Dyslexia

☐ Dyspraxia

16. Do you have any other long-term health problems, conditions or disabilities?

◯ Yes

◯ No

If yes, please describe

[ ]

About using digital technology

The following questions ask about the digital technology that you use. *In this questionnaire, by digital technology we mean electronic devices such as phones, computers, tablets, laptops and apps.*

17. Which of the following digital devices do you use (tick all that apply)?

- [ ] Smartphone
- [ ] Tablet
- [ ] Desktop computer
- [ ] Laptop computer
- [ ] Other please specify

18. Which of the following online resources or activities do you use (tick all that apply)?

- [ ] Social Media/ networking
- [ ] Online gaming
- [ ] Online dating
- [ ] Searching for health information online
- [ ] Video calls (eg. Skype, Zoom, Houseparty, Facetime)
- [ ] Online peer support groups
- [ ] Streaming music (e.g. Spotify)
- [ ] Streaming videos (e.g. Youtube, Netflix)
- [ ] Being creative (e.g. photos, arts-based activities)
- [ ] School/ College/ University work
- [ ] Other, please specify

19. Do you ever use digital technology or online resources (such as those listed above) to help with your mental health or mental well-being?

- ○ Yes
- ○ No

If no, please go to question 21

20. If yes, please tell us the main resources you use and describe how you use them. Include the names of websites, platforms or apps where possible

Adolescence, Digital Technology and Mental Health: young people survey

Digital technology use and COVID-19

21. Has your use of digital technology changed since COVID-19 and lockdown?

- ◯ Yes
- ◯ No

If yes, please outline how it has changed.

22. Have you used digital resources in any of the following ways to help with your mental well-being during COVID-19 and lockdown? (Tick all that apply)

- ☐ To keep in touch with friends/ family
- ☐ To blog about your feelings/ experiences
- ☐ To express feelings/ seek help from others (eg. on apps, social media)
- ☐ To receive remote therapy in place of usual face-to-face services
- ☐ To participate in social networking
- ☐ To find out information about symptoms
- ☐ To find self-help tools (eg. mood monitoring apps)
- ☐ To find advice or suggestions for self-care
- ☐ To join online support groups or peer support
- ☐ Other (please describe)

23. Do you expect to continue to use digital technology in any of the ways described above after COVID?

- ◯ Yes
- ◯ No
- ◯ If yes, please describe which.

24. If you have received online therapy **due to COVID**, please describe your experiences (including likes and dislikes)

Adolescence, Digital Technology and Mental Health: young people survey

Views on digital technology use and mental health

Please indicate how much you agree with the following statements

25. Social media has been helpful to my mental health

- Strongly agree
- Agree
- Neither agree nor disagree
- Disagree
- Strongly disagree
- Unsure

26. Social media has been harmful to my mental health

- Strongly agree
- Agree
- Neither agree nor disagree
- Disagree
- Strongly disagree
- Unsure

27. I am aware of mental health apps for young people

- Strongly agree
- Agree
- Neither agree nor disagree
- Disagree
- Strongly disagree
- Unsure

28. I can find help for my mental health online

- Strongly agree
- Agree
- Neither agree nor disagree
- Disagree
- Strongly disagree
- Unsure

29. I have had bad experiences online that have affected my mental health

- Strongly agree
- Agree
- Neither agree nor disagree
- Disagree
- Strongly disagree
- Unsure

Adolescence, Digital Technology and Mental Health: young people survey

Digital technology and health care

The next questions focus on talking about or using digital technology with professionals who care for young people's mental health and wellbeing.

30. Have you ever received help with your mental health from a GP or someone working in mental health services (e.g. a doctor, nurse, psychologist or occupational therapist)?

- Yes
- No

If no, please move to question 44

31. Have you <u>ever</u> been asked about your digital technology use by any of the following? (Tick all that apply)

- ☐ Doctor (GP)
- ☐ Mental health nurse or doctor
- ☐ Counsellor
- ☐ Youth worker
- ☐ Helpline advisor
- ☐ Social worker
- ☐ Teacher/Tutor
- ☐ Other (please specify)

If you did not tick anyone, or suggest an ‘other’, move to question 34

32. Which topics have you been asked about during these conversations? (tick any that apply)

	Who asked you about this topic?
Use of social media and social networking	
Online gaming	
Online help-services/ signposting	
Use of Mental Health apps	
Participation in chatrooms/ forums	
Negative online experiences	
Online peer support	
Content that you have searched for or browsed online	
Stopping or having a break from use (e.g. using a social media platform)	

Other (please specify)

33. Please tell us if you found it helpful to talk about your digital technology use; **or** explain if it was not helpful.

Sometimes digital technology is used by health professionals when they are helping people with mental health difficulties. Please tick below to show whether any of the following have been part of your care, and which (if any) you would like to happen.

34. Being recommended a help site or other website I could use

- This has happened
- I would like this to happen
- I would not like this to happen

35. Being recommended a mental health app I could use

- This has happened
- I would like this to happen
- I would not like this to happen

36. Being asked to answer questions about how I'm feeling on a device like an iPad during a consultation

- This has happened
- I would like this to happen
- I would not like this to happen

37. Being asked to share what I have posted about my feelings (e.g. on social media or on an app)

- This has happened
- I would like this to happen
- I would not like this to happen

38. Being asked to share my data from a mental health app, such as a mood monitoring tool.

- This has happened
- I would like this to happen
- I would not like this to happen

39. Meeting a health professional online before my first face-to-face appointment with them

- This has happened
- I would like this to happen
- I would not like this to happen

40. Having an appointment online instead of face-to-face

- This has happened
- I would like this to happen
- I would not like this to happen

41. If you ticked to show that any of the above has happened, please tell us which were helpful to you, and how.

42. If you ticked to show that any of the above has happened, please tell us which were unhelpful to you, and how.

43. Please use this space if you wish to comment further on any of the suggestions given above, or to provide other examples that have not been covered.

About digital technology, mental health and health care

Please indicate how much you agree with the following statements

44. I would like to use digital technology as part of my mental health care

- Strongly agree
- Agree
- Neither agree nor disagree
- Disagree
- Strongly disagree
- Unsure

45. It is not appropriate for a health professional to ask a young person about what they do online

- Strongly agree
- Agree
- Neither agree nor disagree
- Disagree
- Strongly disagree
- Unsure

46. I would find it okay to discuss my digital technology use with a health professional

- Strongly agree
- Agree
- Neither agree nor disagree
- Disagree
- Strongly disagree
- Unsure

47. I would feel comfortable sharing mental health app data with a health professional who could help me with my mental health

- Strongly agree
- Agree
- Neither agree nor disagree
- Disagree
- Strongly disagree
- Unsure

48. Most health professionals do not understand the way young people use the online world

- Strongly agree
- Agree
- Neither agree nor disagree
- Disagree
- Strongly disagree
- Unsure

49. Health professionals should have a role in helping to keep young people safe online

- ◯ Strongly agree
- ◯ Agree
- ◯ Neither agree nor disagree
- ◯ Disagree
- ◯ Strongly disagree
- ◯ Unsure

Adolescence, Digital Technology and Mental Health: young people survey

Research

50. What do you think are the most important topics we should research about young people's digital technology use and mental health?

If you would like to be contacted about any further research we do on this subject, please provide your contact details here

If you would like to enter a free prize draw to win a £50 voucher, please provide your contact details here

Please note, your contact details will not be linked to your questionnaire responses.

Thank you for taking the time to complete this questionnaire.
