# Supplementary material for: Digital Technology Use and Mental Health Consultations: Survey of the Views and Experiences of Clinicians and Young People
Source: JMIR Ment Health. 2023 Apr 17;10:e44064. doi: 10.2196/44064 (PMC10152330; doi:10.2196/44064)
Supplement: Multimedia Appendix 2 [file mental_v10i1e44064_app2.pdf]

Adolescence, Digital Technology and Mental Health: practitioners survey

Participant Information and Consent Statement

What is the purpose of the study?

Digital technology can present both opportunities and harms to young people and their mental health. For example, technology can be used to deliver therapeutic interventions or to interface with services to provide additional means of building relationships with and supporting service users. Equally, heavy technology use amongst young people has been linked to poor mental health outcomes and may pose specific risks for those already experiencing difficulties such as self-harm, suicidal ideation or eating disorders. There has been some suggestion that such risks could be considered during mental health consultations. Our research will work with key stakeholders, including health and social care practitioners, to explore questions around how young people’s digital technology use could be drawn upon and responded to during mental health consultation to improve the delivery of care and to manage risk. As a first step, we want to scope current practice, including that which is informal or innovative; and to gather practitioners' views about what might be beneficial, feasible and acceptable.

Before completing this survey, please read the participant information sheet which can be found here.

* 1. Before beginning, please tick to confirm the following:

- [ ] I confirm that I am a practicing health or social care practitioner
- [ ] I have read the participant information sheet
- [ ] I agree to take part in this survey and for my anonymised data to be processed for research purposes by the University of Bristol.

If you have any questions, please contact Dr Lucy Biddle: lucy.biddle@bristol.ac.uk or Dr Jane Derges: jane.derges@bristol.ac.uk

Adolescence, Digital Technology and Mental Health: practitioners survey

About your role

2. Which sector do you work in? (Eg. Statutory, private, voluntary/ third sector)?

____

3. For approximately how long have you worked within health or social care?

Years __________

Months __________

4. What is your current job title? (Eg. General Practitioner, CAMHS nurse, Counsellor)

____

5. For how long have you worked in your current role?

Years

Months

6. Approximately what proportion of your clinical time is spent supporting young people (up to 24years old) with their mental health through face-to-face contact? (Tick one option)

- ○ 0-25%
- ○ 26-50%
- ○ 51-75%
- ○ 76-100%

7. To which of the following age groups do you provide mental health support? (Tick all that apply)

- ☐ Under 12 years
- ☐ 12 – 15 years
- ☐ 16 – 17 years
- ☐ 18 – 24 years
- ☐ Other (please specify)

8. Do you provide support to any specific groups of young people as all or part of your caseload? (Eg. those with neurodevelopmental disorders such as autism, or individuals from minority groups)?

- ○ Yes
- ○ No

If yes, please specify

9. What are the main mental health problems/ diagnoses you support young people with? (Eg. anxiety, depression, self-harm, eating disorder, social problems, suicidal thoughts, trauma).

10. In which country do you currently practice/ work? (If UK, please specify England, Scotland, Wales, Northern Ireland)

Adolescence, Digital Technology and Mental Health: practitioners survey

The following questions ask about your views on young people's use of digital technology in relation to their mental health. *__Here we are using the term digital technology to refer to electronic devices that generate, store or process data. Well known examples include social media, online games, apps and mobile phones.__*

Adolescence, Digital Technology and Mental Health: practitioners survey

Views on young people's digital technology use and mental health

Please indicate how much you agree with the following statements

11. Overall, I have a good understanding of how young people use digital technology

12. I am aware of a range of mental health apps young people could use if experiencing mental health difficulties

13. Social media can be used effectively to provide peer support for young people

14. Young people's digital technology use can be helpful to their mental health

15. Young people's digital technology use can be harmful to their mental health

Adolescence, Digital Technology and Mental Health: practitioners survey

Integrating technology and practice

The following section asks about *__ways that digital technology use could be incorporated into, or considered within, your practice when supporting young people with mental health difficulties__*. This can include a wide range of things, for instance: asking a patient about their Google search history, using iPads to collect outcome data, recommending a mood monitoring app, or delivering therapy online.

16. Have you ever used digital technology to support, complement or enrich mental health consultations with young people?

- ◯ Yes
- ◯ No

If yes, please state how (please indicate any new activity due to COVID-19)

17. Do you currently use digital technology in any way to support the assessment of young people with mental health difficulties?

- ◯ Yes
- ◯ No

If yes, please describe how (please indicate any new activity due to COVID-19)

18. Do you currently use digital technology in any way to support the treatment of young people with mental health difficulties?

- ◯ Yes
- ◯ No

If yes, please describe how (please indicate any new activity due to COVID-19)

19. Do you currently use digital technology in any way to support monitoring the progress of young people with mental health difficulties?

- ◯ Yes
- ◯ No

If yes, please describe how (please indicate any new activity due to COVID-19)

20. If you have described activities in questions 16 -19 above: Have you evaluated any of the activities described?

Yes/No	
If yes, please provide details	
In your experience, were the activities described beneficial? Please explain.	

Adolescence, Digital Technology and Mental Health: practitioners survey

Please indicate which of the following you would consider incorporating into your contacts with young people.

21. Meeting a young person online before face-to-face meeting

- ◯ I already do this
- ◯ I would consider this
- ◯ I wouldn't consider this
- ◯ Unsure

22. Offering therapy online instead of face-to-face (unrelated to COVID restrictions)

- ◯ I already do this
- ◯ I would consider this
- ◯ I wouldn't consider this
- ◯ Unsure

23. Prescribing a self-care or mood monitoring app

- ◯ I already do this
- ◯ I would consider this
- ◯ I wouldn't consider this
- ◯ Unsure

24. Asking a young person to share their mental health app data with me

- ◯ I already do this
- ◯ I would consider this
- ◯ I wouldn't consider this
- ◯ Unsure

25. Viewing/ discussing a young person’s app data to help with history-taking

- [ ] I already do this
- [ ] I would consider this
- [ ] I wouldn't consider this
- [ ] Unsure

26. Viewing/ discussing a young person’s app data to inform triage/ risk assessment

- ◯ I already do this
- ◯ I would consider this
- ◯ I wouldn't consider this
- ◯ Unsure

27. Viewing/ discussing a young person’s app data to help with monitoring of symptoms/ mood

- ◯ I already do this
- ◯ I would consider this
- ◯ I wouldn't consider this
- ◯ Unsure

28. Please use this space if you wish to comment on any of these items, or to provide further examples of integrating technology and practice, which have not been covered.

Talking to patients about risky digital technology use and its impact on mental health

The following questions focus on whether in your role as a practitioner you ever ask young people about the ways that they use digital technology, which could be harmful to their mental health. For instance, this might include asking about their use of social media, participation in chatrooms, or use of the internet to research topics relating to mental health.

29. At your place of work, during mental health consultations, is digital technology use routinely discussed with young people? (eg. integrated into mental health assessment)

- ◯ Yes
- ◯ No

30. Have you received any training/ guidance for talking to young people about their digital technology use?

- ◯ Yes
- ◯ No
- ◯ If yes, please describe briefly

31. Do you have access to a protocol to guide discussion of digital technology use with young people?

- ◯ Yes
- ◯ No
- ◯ If yes, please describe briefly

32. Do you <u>ever</u> ask young people about their digital technology use during mental health consultations?

- ◯ Yes
- ◯ No

If no, please go to question 39

33. Do you deliberately discuss digital technology use with young people?

- ◯ Yes
- ◯ No, I only talk about this if it comes up

34. How often in consultations do you discuss digital technology use with young people?

- ◯ Rarely
- ◯ Occasionally
- ◯ Routinely

35. In what situation are you likely to ask a young person about their digital technology use? (Tick all that apply)

- [ ] As part of early intervention
- [ ] During initial mental health assessment
- [ ] During risk assessment
- [ ] During routine consultations
- [ ] When devising treatment plans
- [ ] When devising crisis plans
- [ ] Other (please specify)

36. Are you more likely to discuss digital technology use with particular groups of young people?

- ○ Yes
- ○ No
- ○ If yes, please specify (eg, specific diagnosis/problem; age; sex; other)

37. Which of the following topics have you addressed during conversations with young people about their digital technology use? (tick all that apply)

- [ ] Use of social media and social networking
- [ ] Online gaming
- [ ] Online help-services/ signposting
- [ ] Use of Mental Health apps
- [ ] Participation in chatrooms/ forums
- [ ] Negative online experiences
- [ ] Positive online experiences
- [ ] Online peer support
- [ ] Self-harm related use (eg. exploring images or searching for information)
- [ ] Online gambling
- [ ] Accessing pornography
- [ ] Other (please specify)

38. Please tell us whether and in what ways you have found it beneficial <u>or not</u> to talk about digital technology use during mental health consultations with young people.

39. Have you ever actively adopted an informal safeguarding role in relation to a young person's digital technology use, such as suggesting a social media break?

- ◯ Yes
- ◯ No
- ◯ If yes, please give details

40. What challenges or barriers are there to discussing digital technology with young people in mental health consultations? (Tick any that apply).

- ☐ Experience no barriers
- ☐ Time constraints
- ☐ Young people are reluctant to discuss
- ☐ I do not consider it my remit
- ☐ Asking could affect my rapport with the young person
- ☐ Raising the topic may be risky (eg. make young person aware of harmful resources)
- ☐ I lack sufficient knowledge/ expertise about digital world
- ☐ I would not know how to respond if a young person disclosed harmful use
- ☐ Other (please describe)

Views on integrating digital technology into mental health consultations

Please indicate how much you agree with the following statements

41. Digital technology may provide a useful tool for enriching consultations with young people

42. It is appropriate to discuss digital technology use in mental health-related consultations with young people

43. Exploring digital technology use should form an essential part of mental health risk assessment

44. I am confident talking to young people about their digital technology use

45. I would like training in how to talk to young people about their digital technology use

46. If a young person shared their mental health app data with me, I would feel comfortable discussing this with them

47. Health and social care practitioners should contribute to ensuring the safety of young people online

48. Having access to a young person's mental health app data would be useful to me in my practice

Clinical needs and research priorities

49. Which of the following research areas would be most useful to you in your practice? (please rank, where 1st is most useful).

- How to maximise treatment options by incorporating digital mental health technology
- Provision of well-evaluated mental health apps
- Exploring how therapy can be delivered digitally (eg. video conference appointments, automated CBT)
- Co-created guidance for talking to young people about digital technology use

50. Please list other clinical needs/ research priorities in this area

Adolescence, Digital Technology and Mental Health: practitioners survey

Participation in further work

We would like to conduct online focus group discussions or telephone follow-up interviews with some survey participants to discuss some of the issues contained in the questionnaire in more detail. We are interested to learn about innovative practice as well as more general views. If you are willing to be contacted, please leave your contact details here

If you wish to be added to our network of stakeholders with interest in the area of adolescence and digital mental health, please leave your contact details here

Your contact details will not be linked to your questionnaire responses.

Thank you for taking the time to complete this questionnaire
