# Supplementary material for: Digital Technology Use and Mental Health Consultations: Survey of the Views and Experiences of Clinicians and Young People
Source: JMIR Ment Health. 2023 Apr 17;10:e44064. doi: 10.2196/44064 (PMC10152330; doi:10.2196/44064)
Supplement: Multimedia Appendix 3 [file mental_v10i1e44064_app3.docx]

Table S1: General attitudes and experiences of digital Technology

| **Young Person (YP) or Clinician (C) Sample** | **Question** | % (n) excluding missing | | | Missing  n (% of total participants) |
| --- | --- | --- | --- | --- | --- |
|  |  | **Agree** | **Unsure/Neutral** | **Disagree** |  |
| **Social Media helpful or harmful?** | | | | |  |
| YP | Social media has been helpful to my mental health | 35.9% (102) | 37.0% (105) | 27.1% (77) | 36 (11.3%) |
| C | Young people’s digital technology use can be helpful to their mental health | 82.1% (78) | 15.8% (15) | 2.1% (2) | 4 (4.0%) |
| YP | Social media has been harmful to my mental health | 43.0% (122) | 35.6% (101) | 21.5% (61) | 36 (11.3%) |
| C | Young people’s digital technology use can be harmful to their mental health | 95.8% (91) | 4.2% (4) | 0.0% (0) | 4 (4.0%) |
| **Awareness of online mental health resources** | | | | |  |
| YP | I am aware of mental health apps for young people | 64.4% (183) | 15.1% (43) | 20.4% (58) | 36 (11.3%) |
| C | I am aware of a range of mental health apps young people could use if experiencing mental health difficulties | 81.1% (77) | 12.6% (12) | 6.3% (6) | 4 (4.0%) |
| YP | I can find help for my mental health online | 62.3% (177) | 24.3% (69) | 13.4% (38) | 36 (11.3%) |
| C | Social media can be used effectively to provide peer support for young people | 74.7% (71) | 20.0% (19) | 5.3% (5) | 4 (4.0%) |
| **Bad experiences online** | | | | |  |
| YP | I have had bad experiences online that have affected my mental health | 46.3% (131) | 20.9% (59) | 32.9% (93) | 37 (11.6%) |
| **Use of digital technology** | | | | | |
|  |  | **Yes** | **No** |  |  |
| YP | Do you ever use digital technology or online resources to support your mental health? | 52.9% (157) | 47.1% (140) |  | 23 (7.2%) |

**Table S2**

1. **Ordinal logistic regression of views on “Social media has been helpful to my mental health”**

**Pseudo R^2^ = 0.05**

**N = 283**

|  | Proportional Odds Ratio | P value | 95% Confidence Interval |
| --- | --- | --- | --- |
|  |  |  |  |
| **Gender (cf. Female)** |  |  |  |
| Male | 2.19 | 0.002 | 1.34 to 3.58 |
|  |  |  |  |
| **Sexuality (cf. Heterosexual)** |  |  |  |
| LGB+ | 2.24 | 0.002 | 1.33 to 3.75 |
|  |  |  |  |
| **Age Group (cf. < 16)** |  |  |  |
| 16-18 | 1.1 | 0.757 | 0.62 to 1.95 |
| >18 | 0.49 | 0.009 | 0.29 to 0.84 |

**Higher odds represent a higher chance of responding positive on a 5 point Likert scale.**

1. **Ordinal logistic regression of views on “Social media has been harmful to my mental health”**

**Pseudo R^2^ = 0.09**

**N = 283**

|  | Proportional Odds Ratio | P value | 95% Confidence Interval |
| --- | --- | --- | --- |
|  |  |  |  |
| **Gender (cf. Female)** |  |  |  |
| Male | 0.31 | <0.001 | 0.19 to 0.51 |
|  |  |  |  |
| **Sexuality (cf. Heterosexual)** |  |  |  |
| LGB+ | 0.76 | 0.31 | 0.45 to 1.29 |
|  |  |  |  |
| **Age Group (cf. < 16)** |  |  |  |
| 16-18 | 2.36 | 0.004 | 1.31 to 4.25 |
| >18 | 3.67 | <0.001 | 2.09 to 6.47 |

**Higher odds represent a higher chance of responding positive on a 5 point Likert scale.**

1. **Ordinal logistic regression of views on “I have had bad experiences online that have affected my mental health”**

**Pseudo R^2^ = 0.04**

**N = 282**

|  | Proportional Odds Ratio | P value | 95% Confidence Interval |
| --- | --- | --- | --- |
|  |  |  |  |
| **Gender (cf. Female)** |  |  |  |
| Male | 0.56 | 0.02 | 0.34 to 0.91 |
|  |  |  |  |
| **Sexuality (cf. Heterosexual)** |  |  |  |
| LGB+ | 1.52 | 0.12 | 0.90 to 2.57 |
|  |  |  |  |
| **Age Group (cf. < 16)** |  |  |  |
| 16-18 | 1.32 | 0.35 | 0.74 to 2.36 |
| >18 | 1.68 | 0.061 | 0.98 to 2.88 |

**iv) Ordinal logistic regression of views on “I would find it okay to discuss my digital technology use with a health professional”**

**Pseudo R2 = 0.05**

**N = 264**

|  | Proportional Odds Ratio | P value | 95% Confidence Interval |
| --- | --- | --- | --- |
|  |  |  |  |
| **Gender (cf. Female)** |  |  |  |
| Male | 0.9 | 0.70 | 0.51 to 1.57 |
|  |  |  |  |
| **Sexuality (cf. Heterosexual)** |  |  |  |
| LGB+ | 0.86 | 0.62 | 0.47 to 1.57 |
|  |  |  |  |
| **Age Group (cf. < 16)** |  |  |  |
| 16-18 | 1.62 | 0.16 | 0.83 to 3.18 |
| >18 | 2.05 | 0.027 | 1.09 to 3.88 |

**V) Ordinal logistic regression on “Have you ever been asked about your digital technology use?”**

**Pseudo R2 = 0.09**

**N = 118**

|  | Proportional Odds Ratio | P value | 95% Confidence Interval |
| --- | --- | --- | --- |
|  |  |  |  |
| **Gender (cf. Female)** |  |  |  |
| Male | 0.55 | 0.231 | 0.21 to 1.46 |
|  |  |  |  |
| **Sexuality (cf. Heterosexual)** |  |  |  |
| LGB+ | 2.32 | 0.05 | 1.00 to 5.39 |
|  |  |  |  |
| **Age Group (cf. < 16)** |  |  |  |
| 16-18 | 0.6 | 0.409 | 0.18 to 2.03 |
| >18 | 0.22 | 0.008 | 0.07 to 0.67 |

**VI) Logistic regression on agreement with wanting to share “my data from a mental health app such as mood monitoring” in individuals who had received help for their mental health and had never been asked to share their data.**

**Pseudo R2 = 0.15**

**N = 102**

|  | Proportional Odds Ratio | P value | 95% Confidence Interval |
| --- | --- | --- | --- |
|  |  |  |  |
| **Gender (cf. Female)** |  |  |  |
| Male | 1.95 | 0.24 | 0.64 to 5.96 |
|  |  |  |  |
| **Sexuality (cf. Heterosexual)** |  |  |  |
| LGB+ | 0.96 | 0.924 | 0.38 to 2.43 |
|  |  |  |  |
| **Age Group (cf. < 16)** |  |  |  |
| 16-18 | 1.56 | 0.576 | 0.33 to 7.43 |
| >18 | 10.51 | 0.001 | 2.48 to 44.57 |

**VII) Logistic regression on agreement with wanting to share “what I have posted on social media” in individuals who had received help for their mental health and had never been asked to share their data.**

**Pseudo R2 = 0.13**

**N = 102**

|  | Proportional Odds Ratio | P value | 95% Confidence Interval |
| --- | --- | --- | --- |
|  |  |  |  |
| **Gender (cf. Female)** |  |  |  |
| Male | 3.53 | 0.04 | 1.06 to 11.80 |
|  |  |  |  |
| **Sexuality (cf. Heterosexual)** |  |  |  |
| LGB+ | 1.09 | 0.866 | 0.38 to 3.14 |
|  |  |  |  |
| **Age Group (cf. < 16)** |  |  |  |
| 16-18 | 0.28 | 0.191 | 0.04 to 1.88 |
| >18 | 3.28 | 0.093 | 0.82 to 13.09 |

**Table S3: Discussing digital technology within mental health consultations**

1. **Frequency and Opinions on Discussion of digital technology**

| **Young Person (YP) or Clinician (C) Sample** | **Question** | % (n) excluding missing | | | | Missing  n (% of total participants) |
| --- | --- | --- | --- | --- | --- | --- |
|  |  | **Agree** | **Unsure/Neutral** | **Disagree** | |  |
| **The role of Health Professionals** | | | | | |  |
| **YP** | I would find it okay to discuss digital tech with health professionals | 66.8% (177) | 22.3% (59) | 10.9% (29) | | 55 (17.2%) |
| **C** | Exploring Digital Technology should form an essential part of mental health consultation | 73.5% (61) | 21.7% (18) | 4.8% (4) | | 16 (16.2%) |
| **YP** | It is not appropriate for a health professional to ask a young person about what they do online | 17.0% (45) | 35.5% (94) | 47.6% (126) | | 55 (17.2%) |
| **C** | It is appropriate to discuss digital technology use in mental health related consultations | 94.0% (79) | 4.8% (4) | 1.2% (1) | | 15 (15.2%) |
| **Health Professionals:** **Understanding the online world** | | | | | |  |
| **YP** | Most health professionals do not understand the way young people use the online world | 42.1% (111) | 43.6% (115) | 14.4% (38) | | 56 (17.5%) |
| **C** | Overall, I have a good understanding of how young people use digital technology | 76.8% (73) | 15.8% (15) | 7.4% (7) | | 4 (4.0%) |
| **C** | I am confident talking to YP about technology | 75.0% (63) | 14.3% (12) | 10.7% (9) | | 15 (15.2%) |
| **C** | I would like training in how to talk to young people about their digital technology use | 71.1% (59) | 25.3% (21) | 3.6% (3) | | 16 (16.2%) |
| **Health Professionals:** **Discussing the online world** | | | | | |  |
|  |  | **Routinely** | **Occasionally** | **Rarely** | **Never** |  |
| **C** | How often do you discuss digital technology use with young people | 49.4% (42) | 40.0% (34) | 2.4% (2) | 8.2% (7) | 14 (14.1%) |
|  |  | **Yes** | **No** |  | |  |
| **C** | At your place of work is digital technology use integrated into mental health assessment | 55.8% (48) | 44.2% (38) |  | | 13 (13.1%) |
| **C** | Have you received any training/ guidance for talking to young people about their digital technology use? | 24.4% (21) | 75.6% (65) |  | | 13 (13.1%) |
| **C** | Do you have access to a protocol to guide discussion of digital technology use with young people? | 7.0% (6) | 93.0% (80) |  | | 13 (13.1%) |
| **YP** | Have you ever been asked about your digital technology use? | 60.3% (73) | 39.7% (48) |  | | 51 (29.7%) |

1. **Clinician Reported Barriers to Discussion of digital technology in consultations**

| **What challenges or barriers are there to discussing digital technology with young people in mental health consultations** | **Clinician Questionnaire**  % (n) excluding missing |
| --- | --- |
| Nil | 41.7% (35/84) |
| Time | 34.5% (29/84) |
| Lack of expertise or knowledge | 28.6% (24/84) |
| Young person viewed as likely reluctant | 21.4% (18/84) |
| Concerns raising topic may be risky | 8.3% (7/84) |
| Worries about affecting rapport | 4.8% (4/84) |
| Not within remit | 1.2% (1/84) |
|  |  |
| Missing | N = 15 (15.2%) |

**iii)** Digital Technology Topics discussed within the mental health consultation

| **Young Person (YP) or Clinician (C) Sample** | **Topic** | **Topic Discussed?**  **% (n)**  excluding missing |
| --- | --- | --- |
| YP | Social Media | 71.2% (52/73) |
| C | Social Media | 92.2% (71/77) |
|  |  |  |
| YP | Mental health apps | 67.1% (49/73) |
| C | Mental health apps | 85.7% (66/77) |
|  |  |  |
| YP | Online Signposting | 58.9% (43/73) |
| C | Online help/signposting | 77.9% (60/77) |
|  |  |  |
| YP | Negative online experiences | 47.9% (35/73) |
| C | Negative online experiences | 77.9% (60/77) |
|  |  |  |
| YP | Chatroom Forums | 37.0% (27/73) |
| C | Chatrooms/forums | 51.9% (40/77) |
|  |  |  |
| YP | Stopping or break from use | 37.0% (27/73) |
|  |  |  |
| YP | Gaming Online | 35.6% (26/73) |
| C | Gaming Online | 72.7% (56/77) |
|  |  |  |
| YP | Content searched | 31.5% (23/73) |
|  |  |  |
| YP | Online peer support | 28.8% (21/73) |
| C | Online peer support | 45.5% (35/77) |
|  |  |  |
| C | Self harm related use | 64.9% (50/77) |
|  |  |  |
| C | Positive online experiences | 58.4% (45/77) |
|  |  |  |
| C | Porn | 29.9% (23/77) |
|  |  |  |
| C | Online gambling | 15.6% (12/77) |
| Missing | | |
|  | Clinician | N = 14 (14.1%) |
|  | Young Person | N = 0 |

**Clinicians were not coded as missing if they answered no to the question “Do you ever ask young people about their digital technology use during mental health consultations?” as they would not be expected to answer these questions. Young people were not coded as missing if they answered no to the question “Have you ever received help with your mental health from a GP or someone working in mental health services (e.g. a doctor, nurse, psychologist or occupational therapist)?” as they would not be expected to answer these questions.**

**Table S4 - How can digital technology be used to assist the mental health consultation?**

**(i) *Young Person Sample***

| **Question (only asked to those who reported contact with mental health professionals)** | % (n) excluding missing | | | **Missing (n)** |
| --- | --- | --- | --- | --- |
|  | **Has Happened** | **I would like this** | **I would not like this** |  |
| Meeting online before my first face-to-face | 13.4% (16) | 31.1% (37) | 55.5% (66) | 2 (1.7%) |
| Having an appointment online instead of face-to-face | 21.2% (25) | 18.6% (22) | 60.2% (71) | 3 (2.5%) |
| Sharing my data from a mental health app | 10.2% (12) | 33.1% (39) | 56.8% (67) | 3 (2.5%) |
| Sharing what I have posted about my feelings on social media/app | 8.5% (10) | 19.7% (23) | 71.8% (84) | 4 (3.3%) |
| Recommended help site or other website I could use | 63.9% (76) | 24.4% (29) | 11.8% (14) | 2 (1.7%) |
| Recommended Mental health app I could use | 53.4% (63) | 34.7% (41) | 11.9% (14) | 3 (2.5%) |
| Answer questions on a device during consultation | 23.7% (28) | 28.8% (34) | 47.5% (56) | 3 (2.5%) |
| Question (asked to all) | **Agree** | **Unsure/Neutral** | **Disagree** |  |
| I would feel comfortable sharing mental health app data with a health professional who could help me with my mental health | 64.4% (170) | 22.7% (60) | 12.9% (34) | 56 (17.5%) |
| I would like to use digital technology as part of my mental health care | 48.9% (129) | 37.1% (98) | 14.0% (37) | 56 (17.5%) |

**(ii) Clinician Sample**

| **Question** | % (n) excluding missing | | | |  |
| --- | --- | --- | --- | --- | --- |
|  | **I already do** | **Would consider** | **Wouldn't Consider** | **Unsure** | **Missing n (%)** |
| Meeting a young person online before face-to-face meeting | 36.0% (31) | 50.0% (43) | 3.5% (3) | 10.5% (9) | 13 (13.1%) |
| Offering therapy online instead of face-to-face | 29.9% (26) | 58.6% (51) | 4.6% (4) | 6.9% (6) | 12 (12.1%) |
| Sharing mental health data with me | 8.0% (7) | 72.4% (63) | 6.9% (6) | 12.6% (11) | 12 (12.1%) |
| Viewing data to help with history taking | 5.6% (5) | 76.4% (68) | 3.4% (3) | 14.6% (13) | 14 (14.1%) |
| Viewing data to help with triage/risk assessment | 3.4% (3) | 66.7% (58) | 11.5% (10) | 18.4% (16) | 12 (12.1%) |
| Viewing data to help with Monitoring | 12.6% (11) | 78.2% (68) | 3.4% (3) | 5.7% (5) | 12 (12.1%) |
| Prescribing a self-care or mood monitoring app | 59.8% (52) | 36.8% (32) | 1.1% (1) | 2.3% (2) | 12 (12.1%) |
|  | **Agree** | **Unsure/Neutral** | **Disagree** |  |  |
| Digital technology may provide a useful tool for enriching consultations with young people | 89.3% (75) | 9.5% (8) | 1.2% (1) |  | 15 (15.2%) |
